# Chronic Spontaneous Urticaria – an evaluation of an indirect immunofluorescence method for detecting anti-mast cell IgG antibodies

**DOI:** 10.1186/1710-1492-10-S1-A2

**Published:** 2014-03-03

**Authors:** Bahar Bahrani, Natasha Gattey, Peter Hull

**Affiliations:** 1 Department of Medicine, University of Saskatchewan, Saskatoon, Saskatchewan, Canada, S7N 0W8

## Background

An autoimmune basis is believed to be responsible for about half of all cases of chronic spontaneous urticaria (CSU) with specific IgG antibodies directed at the high affinity receptor sites for IgE on the mast cell. The autologous serum skin test (ASST) is used to identify this autoimmune form of CSU. Currently, basophil histamine release assay and basophil activation test (BAT) have been used as an alternative to the ASST. These tests are not widely available and are limited in that they only provide evidence that the patient’s serum is capable of inducing basophil degranulation.

We have developed an indirect immunofluorescence method to demonstrate the presence of anti-mast cell antibodies using skin sections from a patient with severe bullous mastocytosis.

## Methods

Sections were cut from paraffin embedded blocks from skin biopsied infant with bullous mastocytosis. An EDTA buffer solution for heat-induced epitope retrieval was used. Serum from 69 patients with CSU was used, and fluorescein conjugated human IgG was used to label fixed antibody. An ASST had been previously performed on 66 of the patients with severe urticaria and was found to be positive in 45.45%. 27 of these patients were receiving intravenous immunoglobulin (IVIG) or had received in the past.

## Results

A positive indirect immunofluorescence was found in half the patients. It was positive in 22.73% of the patients with a positive ASST, but was also positive in 25.76% with a negative ASST. The sensitivity and specificity of ASST were calculated to be 46.88% and 52.94%, respectively. We considered the possibility that the use of IVIG might interfere with indirect immunofluorescence, and this subset was omitted giving a sensitivity and specificity of 34.62% and 77.27%, respectively.

## Conclusion

Positive indirect immunofluorescence was found in half the patients with CSU. When IVIG treated patient were excluded the ASST was associated with is a high specificity but with low sensitivity. Indirect immunofluorescence should be considered as better indicator of the autoimmune form of urticaria.

